# Using CRISPR-Cas9 to generate semi-dwarf rice lines in elite landraces

**DOI:** 10.1038/s41598-019-55757-9

**Published:** 2019-12-13

**Authors:** Xingming Hu, Yongtao Cui, Guojun Dong, Anhui Feng, Danying Wang, Chunyan Zhao, Yu Zhang, Jiang Hu, Dali Zeng, Longbiao Guo, Qian Qian

**Affiliations:** State Key Laboratory of Rice Biology, China National Rice Research Institute, Hangzhou, 310006 China

**Keywords:** Plant sciences, Plant breeding, Transgenic plants

## Abstract

Genetic erosion refers to the loss of genetic variation in a crop. In China, only a few original landraces of rice (*Oryza sativa*) were used in breeding and these became the primary genetic background of modern varieties. Expanding the genetic diversity among Chinese rice varieties and cultivating high-yielding and high-quality varieties with resistance to different biotic and abiotic stresses is critical. Here, we used the clustered regularly interspaced short palindromic repeats (CRISPR)/CRISPR-associated protein9(Cas9) genome editing system to edit *Semi-Dwarf1* (*SD1*) in the elite landraces Kasalath and TeTePu (TTP), which contain many desired agronomic traits such as tolerance to low phosphorous and broad-spectrum resistance to several diseases and insects. Mutations of *SD1* confer shorter plant height for better resistance to lodging. Field trials demonstrated that the yield of the new Kasalath and TTP mutant lines was better than that of the wild type under modern cultivation and that the lines maintained the same desirable agronomic characteristics as their wild-type progenitors. Our results showed that breeding using available landraces in combination with genomic data of different landraces and gene-editing techniques is an effective way to relieve genetic erosion in modern rice varieties.

## Introduction

Genetic erosion was proposed by Harlan^1^ in 1975 to describe the genetic resources after the Green revolution, which involved directional selection of semi-dwarf genes in crop plants. The semi-dwarf genes improved plant architecture in response to heavy use of nitrogen fertilizer^2,3^. With the modernization of agriculture and rapid urbanization, only a few modern varieties are now cultivated, in contrast to the large number of landraces used in rice production prior to the 1980s^4^. This has not only led to a genetic bottleneck, but has also intensified the permanent loss of many landraces^5,6^.

In China, the genetic erosion in rice is even more serious due to three main reasons: (1) from 1930–1960, several rice landraces (such as NanTe and Shenglixian) were dominant in the main rice-producing region of the Yangtze rice valley. (2) From 1950–1970, spontaneous *Semi-Dwarf1* (*SD1*) mutant alleles (*sd1*) were derived from the main cultivars^7^, such as NanTe and Zhaiyeqing, or from local Taiwan varieties Dijiaowujian and Aizaizhan, and then were backcrossed into a few main cultivars. The resulting plants underwent heavy selection at the *sd1* locus as well as other loci to give decreased plant height for better lodging resistance and adaptation to high Nitrogen fertilizer condition and gaining high harvest index. The main cultivars selected in the 1930s–1950s contained the *sd1* mutant alleles to confer shorter plants and were used as the main breeding materials for new varieties^8,9^. (3) From 1970 to today, hybrid rice varieties are used to take advantage of the heterosis between two genetically distant rice varieties with semi-dwarf plant architecture and to enhance nitrogen utilization. However, increases in yield have been limited since the late 1990s. In addition, pedigree analysis showed that the parents of the hybrids had similar allelic variations and many of the same traceable linkage blocks, which when combined, may not improve yield- and resistance-related traits^10,11^. Together, these activities resulted in a very narrow genetic pool in the main modern Chinese rice varieties.

Relieving genetic erosion and improving the yield of modern rice varieties to satisfy food supply demands and foster sustainable development is an urgent issue. In our study, we used a clustered regularly interspaced short palindromic repeats (CRISPR)/CRISPR-associated protein 9 (Cas9) genome editing technique to edit *Semi-Dwarf1* (*SD1*) and *Photosensitivity*5 (*SE*5) in the elite landraces Kasalath and TTP (TeTePu), which contain many desirable agronomic traits such as tolerance to low phosphorous^12^ and broad-spectrum resistance to diseases and insects^13^. Our results showed that precise targeting of *SD1* for gene editing in Kasalath or TTP resulted in new lines with a semi-dwarf plant architecture, which is desired in modern rice varieties, and maintained most of the desired agronomic traits of their progenitors. We show that using gene editing on available landraces can rapidly increase genetic diversity and produce new varieties that satisfy current production requirements.

## Results

### Kasalth and TTP *sd1* mutants are resistant to lodging and show good field performance

**Figure 1:**
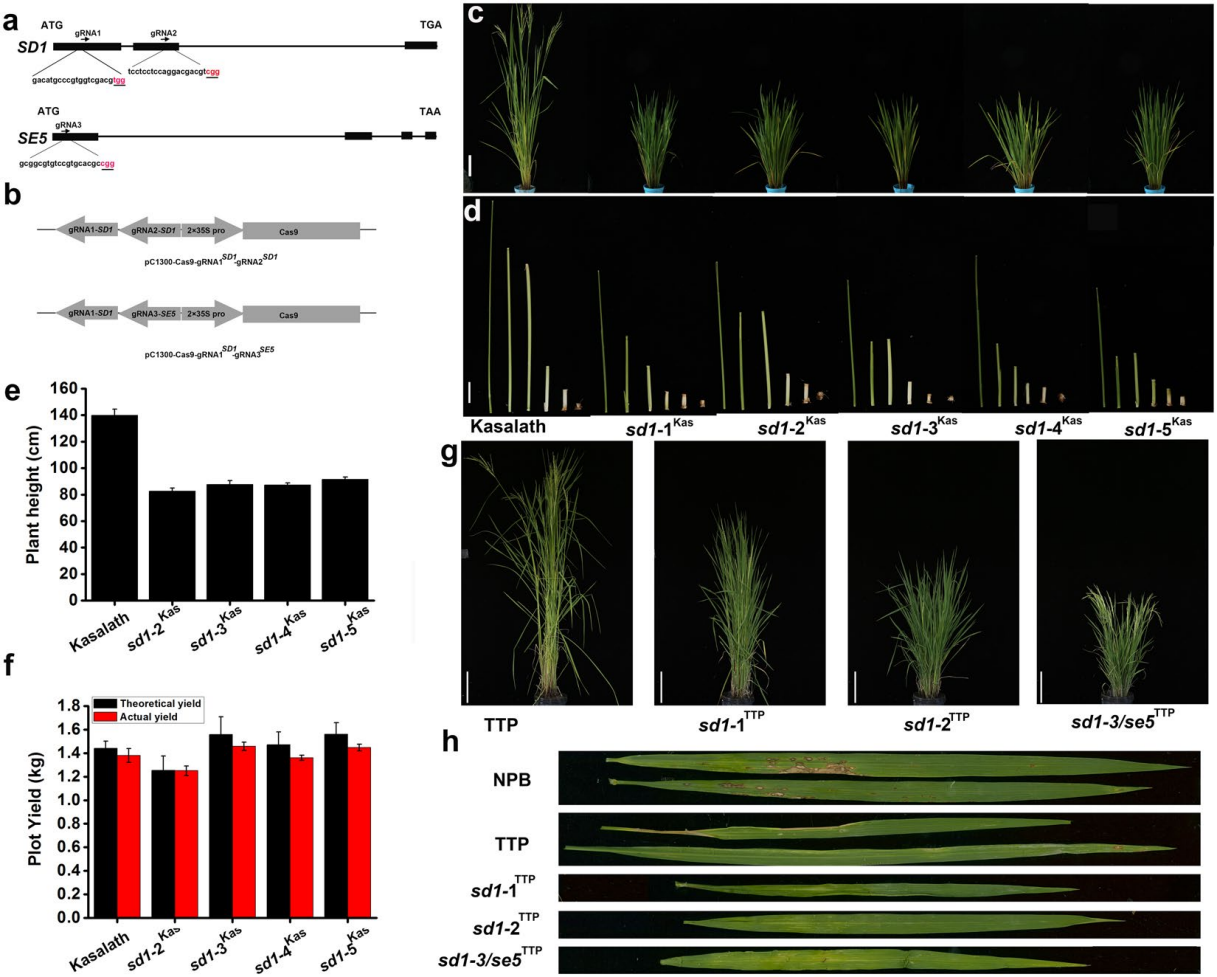
Phenotypes of *sd1* and *se5/ sd1* mutants created by CRISPR-Cas9 in the Kasalath and TTP backgrounds. (**a**) *SD1* and *SE5* target loci. The gRNA1 and gRNA2 targeting sites were designed in the first and second exons of *SD1*, and the gRNA3 targeting site was in the first exon of *SE5*. The target sites are labeled in black lowercase letters. The protospacer adjacent motif (PAM) sequences are underlined and in red type. (**b**) The two vectors used to tranform rice. gRNA1 and gRNA2 were assembled into the pC1300-Cas9 expression vector for Kasalath tranformation; gRNA1 and gRNA3 were assembled into the pC1300-Cas9 expression vector for TTP tranformation. (**c**) Phenotypes of different *sd1* mutants in the Kasalath background. (Bar: 15 cm). (**d**) The internode length proportion in kasalth, *sd1-*1^Kas^, *sd1-*2^Kas^, *sd1-*3^Kas^, *sd1-*4^Kas^, and *sd1-*5^Kas^ mutants. The bar in the image of whole plants represents 4 cm. (**e**) Comparison of plant height among wild type (Kasalath), *sd1-*2^Kas^, *sd1-*3^Kas^, *sd1-*4^Kas^, and *sd1-*5^Kas^. Values in plant height are means±standard deviation (±sd.), n = 10. Bars with different letters are significantly different. Statistical differences among the agronomic traits were detected by Duncan’s multiple range test (p < 0.05). (**f**) Comparison of plot yield (kg of grain per 30 plants) among wild type (Kasalath), *sd1-*2^Kas^, *sd1-*3^Kas^, *sd1-*4^Kas^, and *sd1-*5^Kas^. Bars with different letters are significantly different. Data are means ± standard deviation, n = 3. Statistical differences among the agronomic traits were detected by Duncan’s multiple range test (p < 0.05). (**g**) Phenotypes of *sd1* and *sd1/ se5* mutants in the TTP background. Two *sd1* mutants and one *se5/ sd1* double mutant in TTP. The bar represents 20 cm. **(h**) Diseased leaves of 50- to 60-day-old plants from the field evaluation for resistance to blast: NPB (upper), TTP (upper the second), *sd1* or *sd1/se5* mutants (below). The lesion area of the TTP and TTP mutants were near 0 for all races tested.

To rapidly create new germplasm with decreased plant height in the traditional landraces Kasalath and TTP, we constructed the CRISPR/Cas9 vector for simultaneously targeting *SD1* and *Photoperiod-sensitivity-5* (*SE5*) based on a previous study^14^. The guide RNA (gRNA1 and gRNA2 targeting sites were designed in the first and second exon of *SD1*, and the gRNA3 targeting site was designed in the first exon of *SE5*. The three target sequences (Fig. 1a) were cloned into SK-gRNA, then two gRNAs were assembled into one intermediate vector (SK-gRNA1^SD1^ and SK-gRNA2^SD1^ were assembled into SK-gRNA1^SD1^-gRNA2^SD1^; SK-gRNA1^SD1^ and SK-gRNA3^SE5^ were assembled into SK-gRNA1^SD1^- gRNA3^SE5^). The single guide RNAs (sgRNAs) were cloned into the pC1300-Cas9 expression vector (Fig. 1b).

The binary vectors targeting *SD1* or *SD1* and *SE5* were used for genetic transformation. A total of 25 and 29 positive transgenic plants were obtained in the Kasalath and TTP T_0_ generation, respectively (Supplementary Table 1). We then sequenced the target regions in all positive transgenic plants to analyze the mutations of the target sites. In the gRNA1 and gRNA2 targeting site in Kasalath, the mutation rate was 44 and 68%, respectively. In the gRNA1 and gRNA3 targeting site in TTP, the mutation rate was 28 and 21%, respectively (Supplementary Table 1). This may reveal different editing efficiencies in different genetic backgrounds or accessions.

To eliminate the selective maker gene and T–DNA element, and to evaluate the phenotype of the new *sd1* mutants, T_0_ progeny plants were analyzed by DNA sequencing and self-pollinated to generate T_1_ progeny plants. T_2_ progeny plants that were either homozygous *sd1/sd1* or T-DNA free plants were analyzed by phenotypic screening and genotyping (Fig. S1). Sequencing analysis of Kasalath lines revealed that *sd1-*1^Kas^, *sd1-*2^Kas^, *sd1-*3^Kas^, *sd1-*4^Kas^, and *sd1-*5^Kas^ had a 1-nucleotide (nt), 2-nt, 1-nt, 3-nt, and 61-nt deletions at the predicted editing site, respectively. The *sd1-*1^Kas^ line had a 1-nt deletion next to the gRNA1 protospacer-adjacent motif (PAM), and the *sd1-*4^Kas^ line had a 1-nt deletion next to the gRNA1 PAM and a 2-nt deletion next to the gRNA2 PAM, creating the same stop codon, leading to premature termination. The *sd1-*2^Kas^ line had a 2-nt deletion next to the gRNA2 PAM motif leading to premature termination. The *sd1-*3^Kas^ line had a 1-nt deletion next to the gRNA2 PAM motif leading to premature termination. The *sd1-*5^Kas^ line had a 61-nt deletion next to the gRNA2 PAM motif leading to premature termination (Fig. S2a). All of these mutations would lead to a functionally altered proteins, and have different mutation sites compared with previously reported *sd1* alleles. The previously reported *sd1* spontaneous mutation sites were mostly detected in the gibberellin (GA) 20-oxidase (76-371) motif, varying from a deletion to a single base substitution, indicating that this motif is important for *SD1* function. In our study, the new CRISPR/Cas9 mutation sites were also affected in this region, and the lines showed a dwarf phenotype (Fig. 1c), which supported this hypothesis.

We next evaluated the phenotype of the *sd1* mutants. Although the plant height of the *sd1-*1^Kas^, *sd1-*2^Kas^, *sd1-*3^Kas^, *sd1-*4^Kas^, and *sd1-*5^Kas^ lines was shorter than the WT (Fig. 1c,e), the reduction in internode length was proportional in the mutants compared to the corresponding internode length of the WT (Fig. 1d). As the *sd1-*1^Kas^ and *sd1-*4^Kas^ lines contain the same base deletion at the first editing target, leading to premature transcription termination and a similar mutant phenotype, we chose the *sd1-*2^Kas^, *sd1-*3^Kas^, *sd1-*4^Kas^, and *sd1-*5^Kas^ lines for further research.

To test whether these four allelic variations impact yield, we measured yield-related agronomic traits including plant height, grain number per panicle, etc. The average plant height of the *sd1-*2^Kas^, *sd1-*3^Kas^, *sd1-*4^Kas^, and *sd1-*5^Kas^ lines were 82.5, 87.62, 87.16, and 91.5 cm, respectively (Fig. 1e). Additionally, the grain number per panicle in all the new *sd1* mutants was decreased by various degrees when compared with the WT, and the *sd1-*2^Kas^ line had a significant reduction in the number of spikelets. Conversely, tiller number was notably increased in the mutant lines compared with the WT (Table 1). The heading date was delayed from 3–5 days in the *sd1* mutants (Table 1). For example, the heading date of the *sd1-*2^Kas^ line was delayed by 4.3 days, and the heading date of the *sd1-*5^Kas^ line was delayed by 3 days (Table 1) which showed different effects in the different *sd1* alleles. We calculated the average yield from 30 plants from each of the *sd1-*2^Kas^, *sd1-*3^Kas^, *sd1-*4^Kas^, and *sd1-*5^Kas^ lines. We found that the *sd1-*2^Kas^ line had a strong reduction in yield when compared to the WT and may not be suitable for breeding. The *sd1-*3^Kas^ and *sd1-*5^Kas^ lines showed similar yields to the WT and may be potential breeding materials.

**Table 1:** Agronomic traits comparison between Kasalath and its *sd1* mutant lines.

	Tiller number	Primary branch	Secondary branch	Grain per panicle	Heading date	Spikelet fertiltiy (%)	Grain Weight (g/1000)
Kasalath	13.8 ± 0.4a	9.9 ± 0.5a	41.3 ± 0.8a	233.5 ± 6.8a	84.3 ± 0.6a	83.2 ± 3.5a	18.0 ± 1.1a
*sd1*-2^Kas^	16.1 ± 0.4b	8.9 ± 0.2c	30.7 ± 0.2c	169.6 ± 2.1c	88.3 ± 0.6b	83.1 ± 3.5a	18.4 ± 0.5a
*sd1*-3^Kas^	17.0 ± 0.8b	9.3 ± 0.2bc	36.0 ± 1.6b	199.5 ± 5.5b	87.3 ± 0.6b	81.8 ± 3.8a	18.7 ± 0.4a
*sd1*-4^Kas^	16.7 ± 0.1b	9.4 ± 0.2ab	36.8 ± 0.2b	201.3 ± 7.9b	87.6 ± 0.6b	82.1 ± 1.9a	17.8 ± 1.0a
*sd1*-5^Kas^	16.9 ± 0.9b	9.0 ± 0.1ab	36.1 ± 1.0b	200.5 ± 5.5b	87.3 ± 0.6b	84.8 ± 2.2a	18.1 ± 0.3a

Values in plant height, Grain number per panicle are means ± standard deviation (±sd.), n = 10. Values in tiller number, heading date means ± standard deviation (±sd.), n = 30. thousand grain weight, plot yield trial means ± standard deviation (±sd.), n = 3. Statistical differences among the agronomic traits were detected by Duncan’s multiple range test (p < 0.05).

To study the lines in response to nitrogen fertility, we treated Kasalath and the *sd1-*3^Kas^ and *sd1-*5^Kas^ lines with different concentrations of nitrogen fertilizer in the field. Under high nitrogen, Kasalath is more sensitive than Kasalath *sd1* to nitrogen (Fig. S3a, Table 2). Additionally, Kasalath was more susceptible to lodging than the *sd1-*5^Kas^ line (Figs. S3b, S4). These results suggest that the *sd1* mutation confers better lodging resistance and better nitrogen utilization.

**Table 2:** Yield-related traits comparion between Kasalath and its *sd1* mutant lines under different Nitrogen treatment condition.

		Plant Height	Tiller number	Grain per panicle	Spikelet fertiltiy (%)	Grain Weight (g/1000)	lodging ratio (%)
NN	Kasalath	152.0 ± 2.0a	14.3 ± 0.6a	164.7 ± 4.4a	92.1 ± 6.1a	16.2 ± 0.3a	0
	*sd1*-3^Kas^	107.0 ± 1.0b	17.7 ± 0.6a	139.5 ± 1.3b	85.1 ± 2.2a	16.4 ± 0.3a	0
	*sd1*-5^Kas^	104.7 ± 0.6b	17.7 ± 1.2a	135.7 ± 0.7b	86.3 ± 1.4a	16.7 ± 0.3a	0
LN	Kasalath	152.0 ± 4.4a	27.7 ± 2.3a	152.7 ± 2.6a	80.4 ± 5.3a	17.0 ± 0.1a	0
	*sd1*-3^Kas^	103.7 ± 0.6b	32.3 ± 1.5b	127.6 ± 2.2b	87.3 ± 5.0a	16.0 ± 0.3a	0
	*sd1*-5^Kas^	106.7 ± 2.5b	33.0 ± 3.0ab	156.9 ± 8.7a	81.2 ± 3.0a	16.6 ± 1.0a	0
MN	Kasalath	171.3 ± 4.0a	35.3 ± 4.5a	167.6 ± 6.0a	81.9 ± 2.7a	17.1 ± 0.1a	0.8
	*sd1*-3^Kas^	108.0 ± 2.6b	44.3 ± 2.5b	132.8 ± 2.3b	81.2 ± 3.0a	16.5 ± 0.6a	0
	*sd1*-5^Kas^	105.3 ± 0.6b	49.0 ± 3.6b	141.1 ± 3.3b	85.5 ± 2.8a	16.8 ± 0.2a	0
HN	Kasalath	167.7 ± 5.8a	43.7 ± 3.8a	157.3 ± 7.4a	65.4 ± 2.2a	17.1 ± 0.5a	1
	*sd1*-3^Kas^	105.0 ± 0.0b	45.6 ± 4.9a	126.0 ± 5.7c	72.4 ± 5.1a	16.6 ± 0.6a	0.01
	*sd1*-5^Kas^	109.7 ± 2.1b	42.6 ± 4.9a	138.1 ± 3.6b	73.7 ± 5.0a	17.0 ± 0.6a	0.02

NN: No Nitrogen, LN: Low Nitrogen- 8 kg N ha^−1^, MN: moderate Nitrogen-14 kg N ha^−1^, HN: high nitrogen- 20 kg N ha^−1^. Statistical differences among the agronomic traits were detected by Duncan’s multiple range test (p < 0.05).

Simultaneously, we obtained new *sd1* single mutants and *sd1 se5* double mutants by gene editing of *SD1* and *SE5* in the TTP background carrying no selection marker (Fig. S1b). PCR check SD1 gene of these stable inherited mutant lines confirmed there are different mutation of *SD1* or *SE5* in these corresponding mutant lines (Figs. 1g, S2b). As TTP has broad-spectrum resistance to the rice blast fungus, we inoculated the leaf sheaths of TTP and its mutants with mix of Magnaporthe oryzae strain CH102 and CH184. and found that the *sd1* mutants had slightly increased resistance to rice blast compared with TTP (Fig. 1h). In addition, TTP is a landrace of Vietnam which is a thermo-sensitive and late-flowering in main China rice production area. Knock-out *Se5* gene (*LOC_Os06g40080*) in TTP lead to 2 weeks early flowering than TTP in Hangzhou, China. Overall, using the CRISPR-Cas9 gene editing technique, we obtained new *sd1* mutant alleles in the elite landraces Kasalath and TTP.

### The desirable agronomic traits of the progenitors were maintained in the new *sd1* mutant rice lines

To investigate whether the *sd1* mutation affects the GA response, we tested the GA response of the *sd1-*3^Kas^, *sd1-*4^Kas^, and *sd1-*5^Kas^ lines. Treatment with 1, 10, and 100 mM GA_3_ had similar effects in Kasalath and its mutants (Fig. S5a,c). The effect of different concentrations of GA_3_ on the length of the second internode was also similar between the WT and the mutants (Fig. S5b), indicating that the *sd1-*3^Kas^, *sd1-*4^Kas^, and *sd1-*5^Kas^ lines have a normal sensitivity to GA. Consistently, in GA assay of TTP, *sd1*-1-TTP,*sd1*-2-TTP, we got similar physiological results with *sd1-*3^Kas^, *sd1-*4^Kas^, and *sd1-*5^Kas^ lines (Fig. S6). All these results showed that these sd1mutants attained by Crispr-Cas9 are also GA deficiency mutants as reported those spontaneous *sd1* mutants.

**Figure 2:**
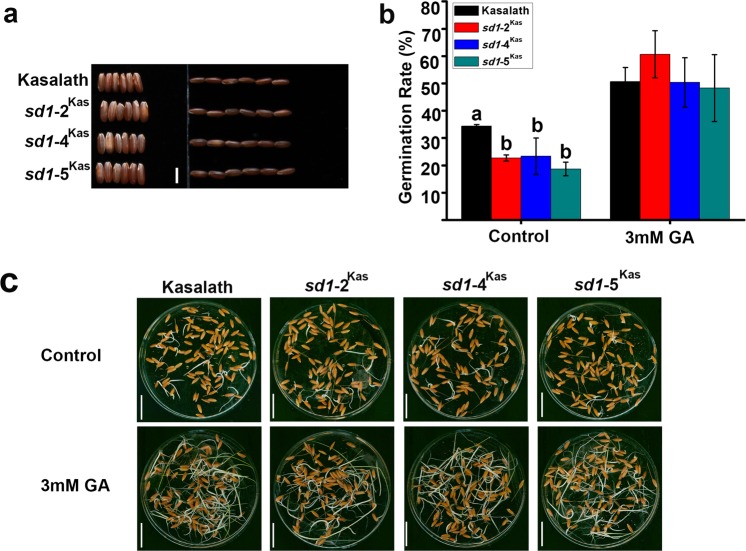
Comparison of germination among Kasalath, *sd1-*2^Kas^, *sd1-*4^Kas^, *sd1-*5^Kas^. (**a**) Images of caryopsis morphologies of Kasalath and the *sd1* mutants. (**b**) Germination rates of Kasalath, *sd1-*2^Kas^, *sd1-*4^Kas^, and *sd1-*5^Kas^ seeds at different concentrations of GA. Germination was evaluated at 6 d after imbibition and with three samples (100 seeds/sample). (**c**) Germination phenotypes of Kasalath, *sd1-*2^Kas^, *sd1-*4^Kas^, *sd1-*5^Kas^. The germination phenotypes are shown at 6 d after imbibition. Values means ± standard deviation (±sd.), n > = 3. Statistical differences were detected by Duncan’s multiple range test (p < 0.05).

Seed dormancy has been reported to be associated with red grain color. Previous studies have shown that Kasalath, which has red grain, contains a cluster of quantitative trait loci at Os07g11020/*Rc* for seed dormancy compared to the white rice cultivar Nipponbare which is 14 bp deletion at *Rc* coding region^15^. An appropriate level of seed dormancy can decrease pre-harvest sprouting, and thus improve grain yield and quality. Therefore, we analyzed whether the *sd1-*2^Kas^, *sd1-*4^Kas^, and *sd1-*5^Kas^ lines have increased seed dormancy like Kasalath (Fig. 2a–c). Seed dormancy was evaluated by measuring the germination of seeds after imbibing for 6 days.

We first sequenced Os07g11020/*Rc* in Nipponbare, Kasalath, and the *sd1-*2^Kas^, *sd1-*3^Kas^, *sd1-*4^Kas^, and *sd1-*5^Kas^ mutants and found a 14-bp deletion in the *rc* from rice variety 93-11 and Nipponbare. Meanwhile, we tested two dormancy-breaking treatments to evaluate the difference in the germination response among Kasalath and its mutants and found that the mutation of *sd1* can, to some degree, increase seed dormancy (Fig. 2b,c), which is similar to findings in a previous study^16^. Together, our results suggest that the *sd1* mutation in Kasalath may decrease the endogenous GA concentration and enhance seed dormancy, which may be beneficial to rice production during harvest under high temperatures and wet conditions.

**Figure 3:**
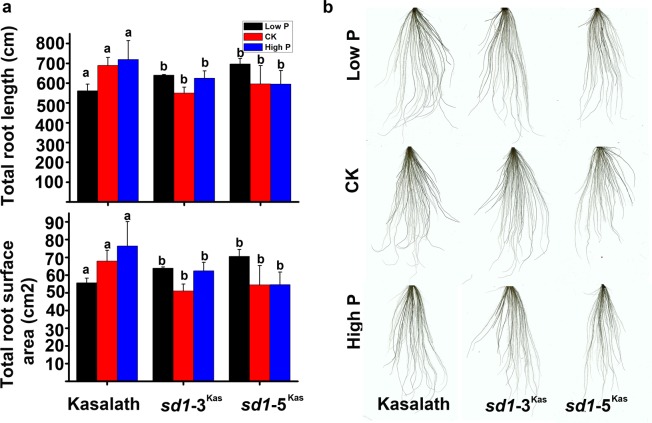
30-day-old of root growth response under different concentration of P. (**a**) Total root length and surface area of Kasalath, *sd1-*3^Kas^, and *sd1-*5^Kas^ in low -P (0.5 mg/L), CK (10 mg/L) and high-P (25 mg/L) hydroponics solution for 20 days. Error bars indicate standard error. (**b**) The root architecture of Kasalath, *sd1-*3^Kas^, and *sd1-*5^Kas^ plants under different concentration of P. Values means±standard deviation (±sd), n > = 3. Statistical differences were detected by Duncan’s multiple range test (p < 0.05).

Phosphorus deficiency has a detrimental impact on plant growth. Phosphatic fertilizers could relieve phosphorus deficiency, but low use efficiency of available phosphorus in rice varieties is a bottleneck and could lead to environmental consequences. Therefore, it is important to use landraces containing genes for high phosphorus use efficiency when breeding new rice varieties. A major quantitative trait locus for tolerance to phosphorus deficiency, *Pup1* (also named *Pstol1*), was identified in Kasalath^12^. This gene is absent from Nipponbare. In order to explore whether the *sd1* mutation in Kasalath impacts phosphorus use efficiency, we conducted phenotypic analyses of Kasalath, *sd1-*3^Kas^, *sd1-*5^Kas^, in nutrient solution with low P (0.5 mg/L), CK (10 mg/L) and high P (25 mg/L) hydroponics solution for 18 days (Fig. 3a,b). Under low P, the root lengths and surface area of *sd1-3*^Kas^, *sd1-5*^Kas^ were increased compare with Kasalath but decline in the CK and high-P concentration (Fig. 3a,b). These results demonstrated that the *sd1* mutation does not disrupt the function of *PSTOL1* in the Kasalath mutants under low P.

Flowering date (also known as heading date) is an important agronomic trait in rice. Photoperiod regulation is an important factor for controlling heading date. *Ghd8*(*Grain Yield, Plant Height and Heading Date8*) is a major quantitative trait locus associated with pleiotropic effects on grain yield, heading date, and plant height^17^. Mutation of *Ghd8* leads to earlier flowering. *Heading date 1* (*Hd1*)^18^ represses flowering under long-day (LD) conditions and induces it under short-day (SD) conditions. Mutants of *Hd1 in* Kasalath exhibited no photoperiod response under LD conditions. Knockout mutants of *OsGSK1* which is an orthologue of Arabidopsis *BIN2* showed enhanced tolerance to cold, heat, salt, and drought stresses^19^. *OsGSK1* in Kasalath contains many single nucleotide polymorphisms (SNPs) and insertions/deletions compared with Nipponbare and 93-11. *S*_*5*_^*n*^ can restore the sterility in *indica* and *japonica* hybrids^20^, which is an important resource when utilizing heterosis between these rice subspecies. *S*_*5*_^*n*^- wide compatible gene exits in Kasalath. In order to test whether the CRISPR-Cas9 editing technique affected *Ghd8, hd1, OsGSK1*, and *S5*^*n*^ and the phenotypic consequences, we sequenced *Rc-HLH*, *Ghd8, hd1*, and *OsGSK* in Kasalath and the *sd1-*2^Kas^, *sd1-*3^Kas^, *sd1-*4^Kas^, and *sd1-*5^Kas^ mutants. In line with previous results, *Rc-HLH* contains a 14-bp insertion in Kasalath and its mutants. *Ghd8* contained the same SNPs and deletion in Kasalath and its mutants. *hd1* contained the same insertion mutation in Kasalath and its mutants. *OsGSK1* contained the same insertion in Kasalath and its mutants. In addition, the loss of function of *S5*^*n*^ was observed in Kasalath and its mutants (Fig. S7).

In the TTP mutant rice lines, we sequenced the *Pi54* gene^21^ which confer high degree of resistance to diverse isolates of *M. oryzae* and found no changes in these rice lines, which is consistent with the resistance test in the field (Fig. S8).All these results show that the *sd1* mutation in Kasalath does not have negative effects on other genes associated with many desirable agronomic traits.

In summary, The CRISPR-Cas9 editing technique can be used to more rapidly create the *sd1* mutation in desirable germplasm. Two *sd1* alleles in Kasalath and one double *sd1 se5* mutant created in this study can serve as potential materials for breeding. Creating additional *sd1* alleles in other desirable landraces could help to improve genetic diversity in rice and benefit rice production.

## Materials and Methods

### Plant materials and Measurements of agronomic traits

The background of transgenic plants is Kasalath or TTP and all the rice plants were grown in the paddy fields under natural conditions in Hangzhou or LingShui (China National Rice Research Institute, China). The agronomic traits were analyze after rice harvested, a total of 10 or 30 randomly chosen rice plant were used to measure plant height, grain number per panicle and tiller number, heading date.

### Plasmid construction and Plant transformation

The three target sites were designed for knock out of *SD1* or *SE5* genes using the CRISPR/Cas9 system. The gRNA1^*SD1*^ (digested with Kpn I/BamH I) and gRNA2^*SD1*^ (digested with Kpn I/Bgl II) were assembled into one intermediate vector. Similar methods were used to assemble gRNA1^SD1^and gRNA3^SE5^ into one intermediate vector. The two intermediate vector (digested with Kpn I/Bgl II) was assembled to the pC1300-Cas9 binary vector (digested with Kpn I/BamH I), respectively. The target sequences are provided in Supplementary Table S2 in Supporting Information. The pC1300-Cas9 binary vector loading two sgRNAs was used for genetic transformation via the Agrobacterium-mediated transformation (strain EHA105) method for generating transgenic rice, according to Japonica rice and Indica rice transformation methods^22,23^. Detection of mutations Genomic DNA of transgenic plants was extracted from approximately 100 mg leaf tissue of rice via the cetyltrimethylammoniumbromide (CTAB) method. PCR was conducted with KOD FX DNA polymerase (Toyobo, Japan) to amplify the fragments surrounding the three target sites. The DNA fragments were sequenced by the Sanger method and analyzed by the degenerate sequence decoding method^24^.

### Seed germination rate measurement

Seed dormancy was evaluated by germination of seed samples after-ripened for 5 days. Harvested rice was dried at 42 °C for 2 days and stored at room temperature before testing. Seeds of Kasalath and T_2_ of the *sd1-*2^Kas^, *sd1-*4^Kas^, *sd1-*5^Kas^ were used in this study. Seed dormancy was evaluated by germination testing. A sample of approximately 100 well-developed seeds was distributed on filter paper in a in 23 cm × 23 cm aseptic culture dishes with 150 ml distilled water at 30 °C and 100% relative humidity. An artificial climate chamber (POX-330B-22H, Life Apparatus Co., Ningbo, China) was used for the seed dormancy treatments. Germination was evaluated visually by protrusion of the radicle from the hull by more than 3 mm and counted daily from day 2 to day 6 or at day 6. A test was replicated three times, and germination percentages were averaged for genetic analysis.

### GA assay on 93-11, Nipponbare, Kasalath and new sd1 mutant lines

Seeds of Kasalath, T_2_ of the *sd1-*4^Kas^, *sd1-*5^Kas^ or TTP*, sd1*-TTP were pre-germinated in the dark at room temperature. After 3 days, germinated seeds pre-germinated in the dark at room temperature were transferred to Yoshida culture solution with 0 mM, 1 mM,10 mM,100 mM GA_3_ respectively. The solution was replaced every 3 days. The plant height and second internode length of seedlings (10 DAG) were analyzed.

### Root scan of Kasalath, *sd1-*3^Kas^, *sd1-*5^Kas^ grown in hydroponics

Seeds of the Kasalath and T_2_ of the *sd1-*3^Kas^, *sd1-*5^Kas^ were pre-germinated in Petri dishes in the dark at room temperature. After 3 days, germinated seeds were transferred to Yoshida culture solution grow for 7 days. Then plants were transferred to Yoshida culture solution with low -P (0.5 mg/L), CK (10 mg/L) and high-P (25 mg/L) respectively^12^. The solution was replaced every 3 days. The root length of seedlings (30 DAG) were checked.

### N fertilizer treatments

Field experiments were conducted in the same field, HangZhou ZheJiang Province, China. The four N treatments were 0 (No N), 8 (Low N), 14 (moderate N) and 20 (high N) kg N ha^−1^. In each treatment, N was applied at the basal, tillering and panicle initiation stages at a ratio of 4: 3: 3. Phosphorus was applied as a basal fertilizer at a rate of 5.6 kg P ha−1 and K was applied equally between the basal and panicle initiation stages at 9.6 kg ha^−1^. Kasalath, *sd1-*3^Kas^, *sd1-*5^Kas^ were used in the N treatments^25^.

### Assessment of disease phenotypes with Magnaportheoryzae

Fifty-day-old of NPB and TTP, *sd1-1*^TTP^, *sd1-2*^TTP^, *sd1-3/se5*^TTP^ plants were inoculated with Magnaporthe oryzae spore suspension (1 × 105 spores/ml). After 7 days post inoculation, disease reaction of each rice line was photoed^26^.

## Supplementary information

Using CRISPR-Cas9 to generate semi-dwarf rice lines in elite landraces
