# Supplementary material for: Using CRISPR-Cas9 to generate semi-dwarf rice lines in elite landraces
Source: Sci Rep. 2019 Dec 13;9:19096. doi: 10.1038/s41598-019-55757-9 (PMC6910903; doi:10.1038/s41598-019-55757-9)
Supplement: Supplementary file 1 — Using CRISPR-Cas9 to generate semi-dwarf rice lines in elite landraces [file 41598_2019_55757_MOESM1_ESM.pdf]

Title: Using CRISPR-Cas9 to generate semi-dwarf rice lines in elite landraces

Xingming Hu#, Yongtao Cui#, Guojun Dong#, Anhui Feng, Danying Wang, Chunyan Zhao, YuZhang, JiangHu, DaliZeng, Longbiao Guo, QianQian*

State Key Laboratory of Rice Biology, China National Rice Research Institute, Hangzhou 310006, China.

# These authors contributed equally to this work.

*Correspondence: qianqian188@hotmail.com

Supplement Information

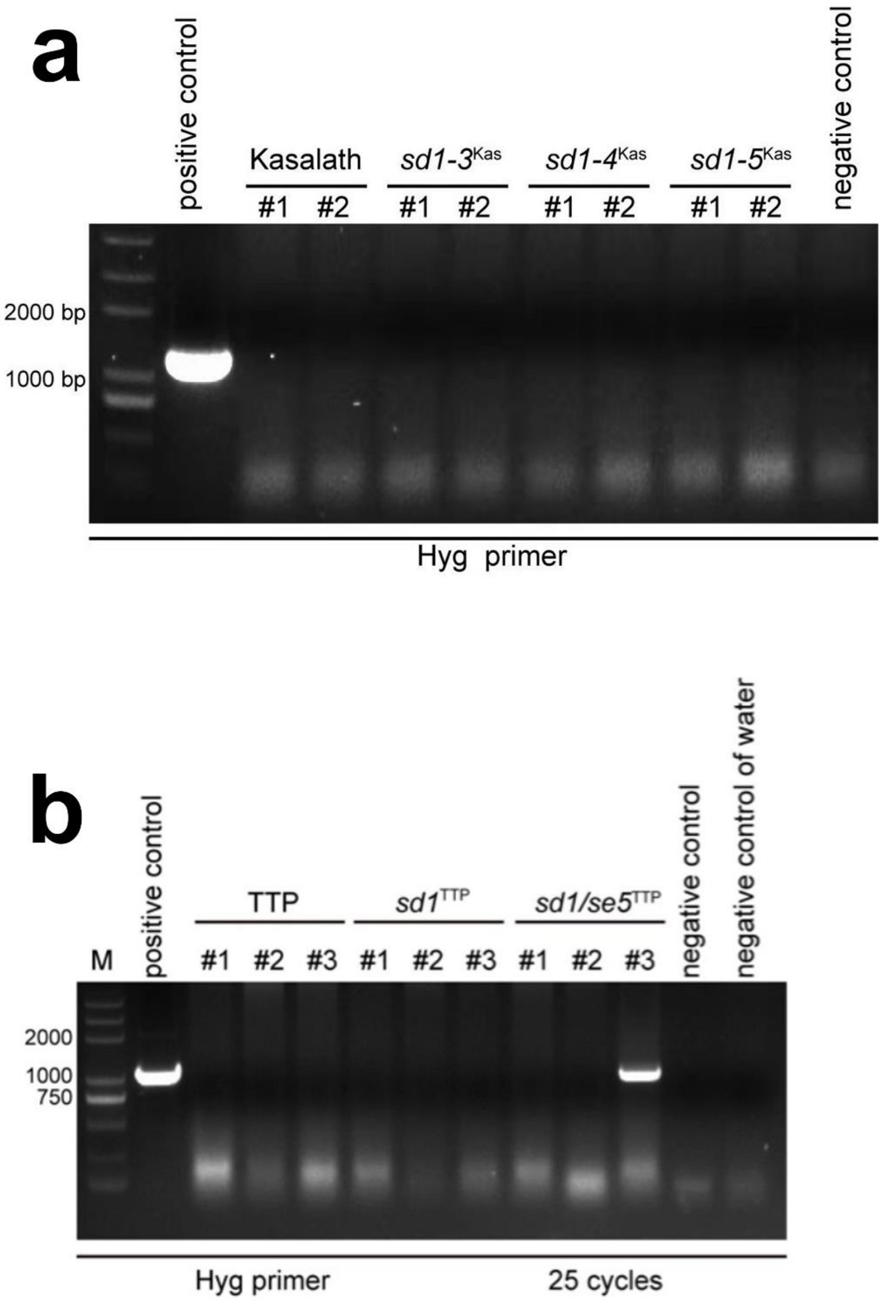

Figure S1: T-DNA free of T2 or T1 progenies of *sd1* mutant alleles derived from Kasalath and TTP (TeTePu) rice were selected by hygromycin identified by PCR. (a) T2 progenies of *sd1-3*^{Kas}, *sd1-4*^{Kas}, *sd1-5*^{Kas} are all T-DNA free lines. (b) T1 progenies of *sd1se5*^{TTP} was found the third line is positive transformant which was removed from further field test.

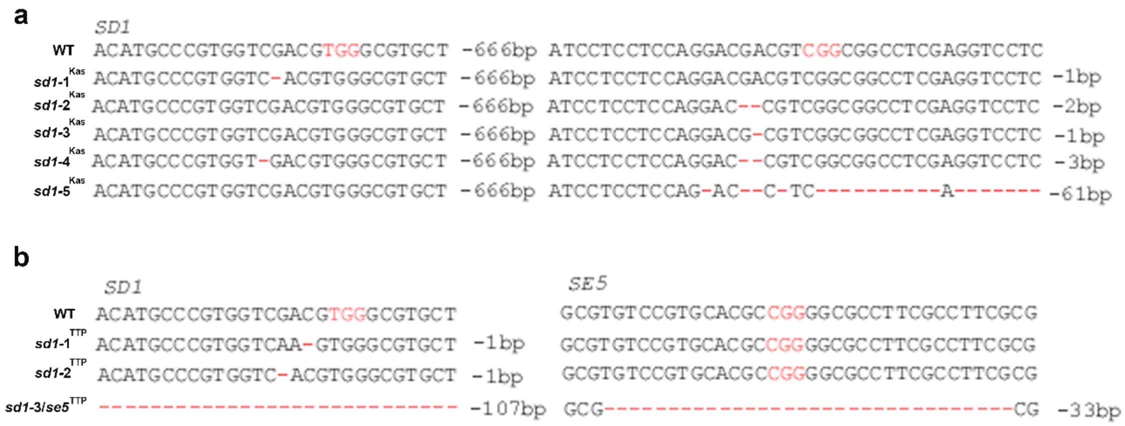

Figure S2: Schematic illustration *sd1* or *se5* mutation sites derived from Kasalath and TTP

(a) Different mutational sites in *sd1* coding sequence region derived from Kasalath by Crispr-Cas9 way

(b) Two *sd1* mutant alleles and double mutant-*sd1se5* derived from TTP by Crispr-Cas9 way

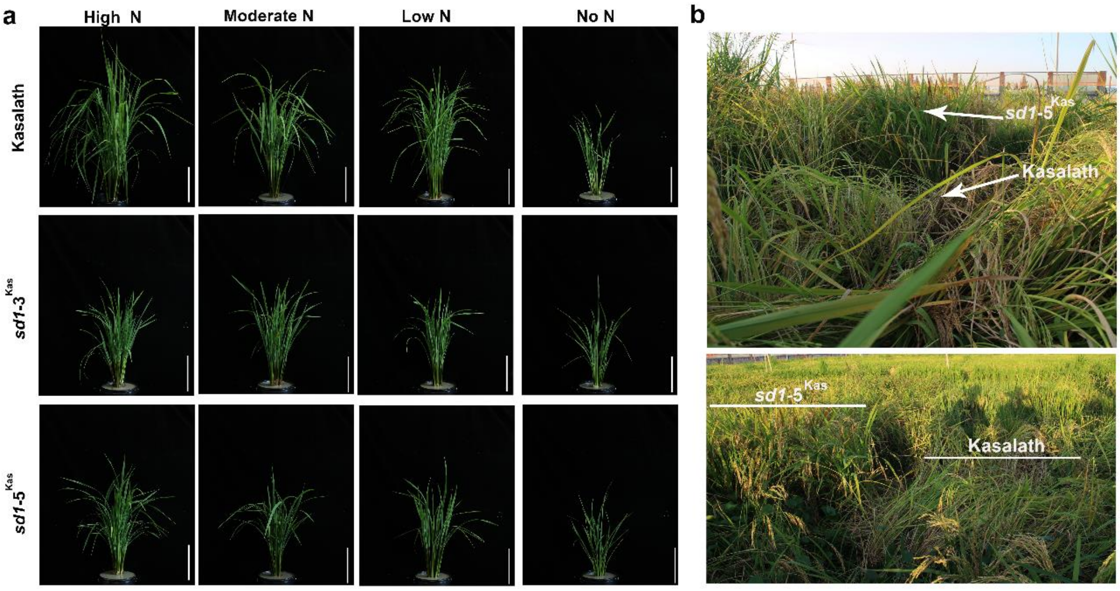

Figure S3: Agronomic traits comparison between Kasalath and its *sd1* mutant lines by Crispr-CAS9 way under different Nitrogen treatment. (a) Plant architecture performance comparison among Kasalath, *sd1-3*^{kas} and *sd1-5*^{kas} under different N-treatment. Bar: 20cm (b) Natural field test show *sd1-5*^{kas} is more obvious lodge-resistance than Kasalath. No N: No Nitrogen, LN: Low Nitrogen-8kg N ha^{-1}, MN: moderate Nitrogen-14 kg N ha^{-1}, HN: high nitrogen-20 kg N ha^{-1}.

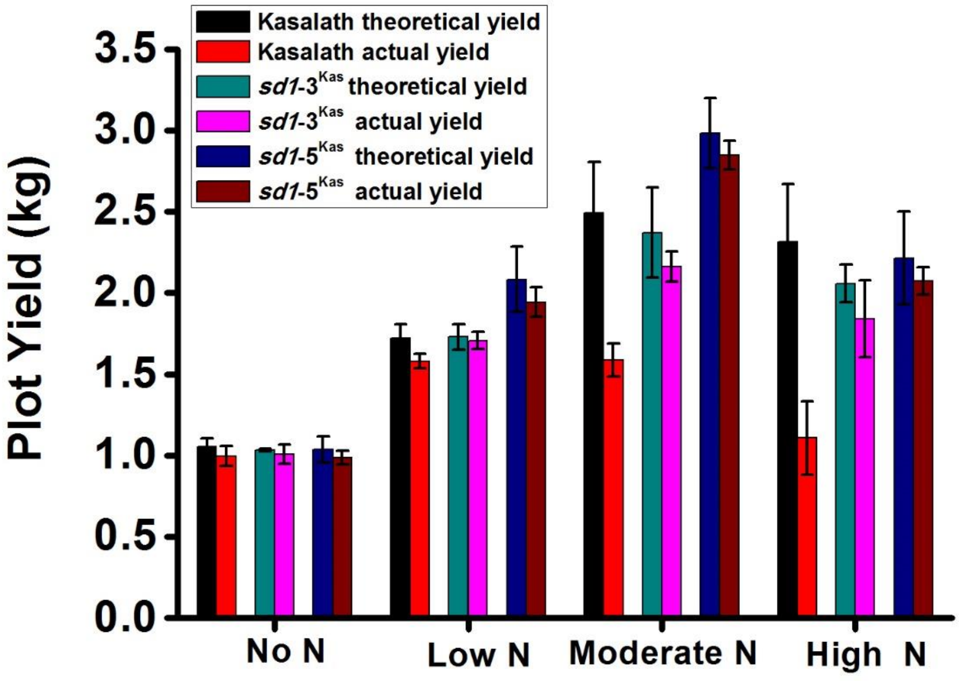

Figure S4: Grain yield comparison among Kasalath and new *sd1* rice lines under field Nitrogen treatment condition.

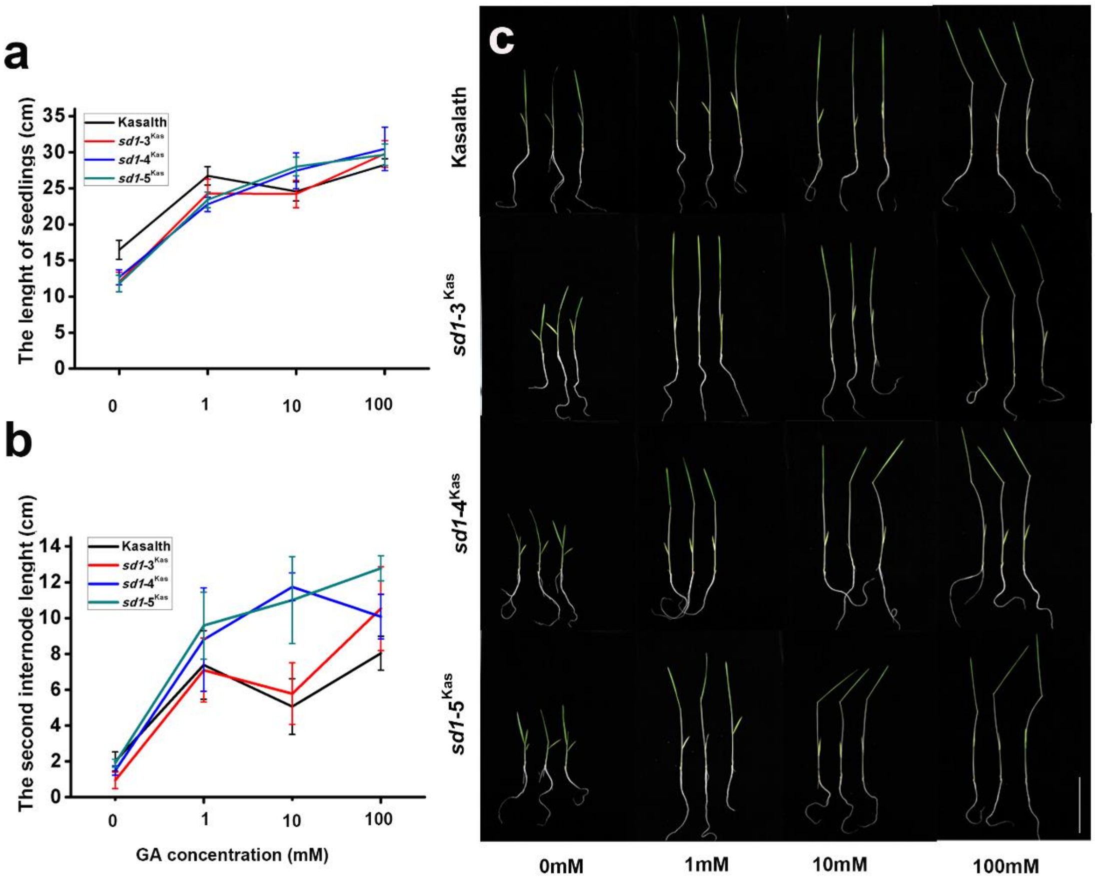

Figure S5: 4-day treatment of *sd1*-3^{Kas}, *sd1*-4^{Kas}, and *sd1*-5^{Kas} with GA3 had similar effects in Kasalath

(a): The length of 7-day-old WT, *sd1*-3^{Kas}, *sd1*-4^{Kas}, and *sd1*-5^{Kas} seedlings at 0, 1, 10, and 100 mM GA.

(b): The second internode length of 7-day-old WT, *sd1*-3^{Kas}, *sd1*-4^{Kas}, and *sd1*-5^{Kas} plants at 0, 1, 10, and 100 mM GA.

(c): 7-day-old of WT, *sd1*-3^{Kas}, *sd1*-4^{Kas}, and *sd1*-5^{Kas} seedlings at 0, 1, 10, and 100 mM GA.

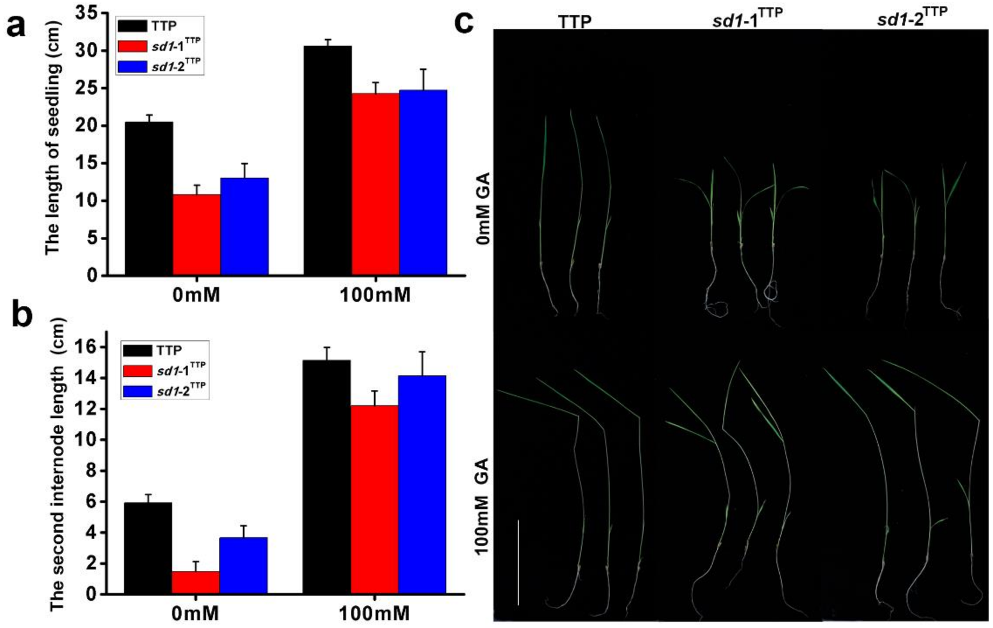

Figure S6: GA deficiency in *sd1-1*^{TTP} and *sd1-2*^{TTP} compared with progenitor-TTP

(a) The length of 7-day-old TTP, *sd1-1*^{TTP}, *sd1-2*^{TTP} seedlings at 0 and 100 mM GA.

(b) The second internode length of 7-day-old TTP, *sd1-1*^{TTP}, *sd1-2*^{TTP} at 0 and 100 mM GA.

(c) 7-day-old of TTP, *sd1-1*^{TTP}, *sd1-2*^{TTP} seedlings

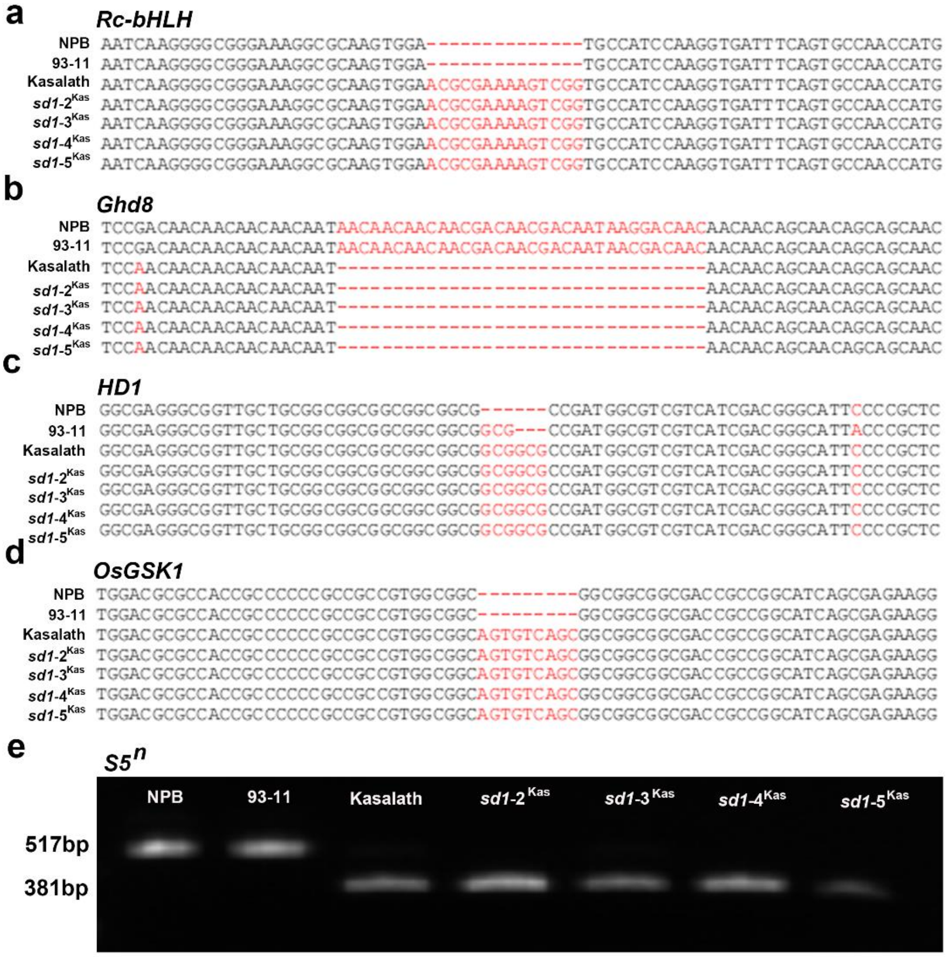

Figure S7: PCR sequence results show good allelic variation in Kasalath maintain new *sd1* mutant lines. (a) *Rc- Os07g0211500*. (b) *Ghd8- Os08g0174500*. (c) *Hd1-Os06g0275000*. (d) *OsGSK1- Os01g0205700*. (e) $S5^{n}$*-Os06g0213100*.

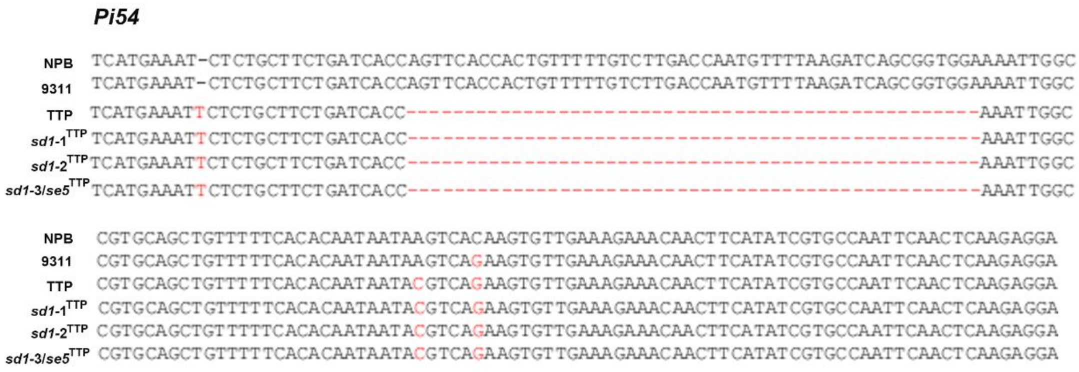

Figure S8: *Pi54* blast resistance gene in TTP keep in new *sd1* mutant lines. *Pi54-Os11g0639100.*

Supplementary Table1: the number of plants detected in the two Kasalath PAM sites and TTP PAM sites

	Kasalath		TTP	
	gRNA1^{SD1}	gRNA2^{SD1}	gRNA1^{SD1}	gRNA3^{SE5}
Number of plants detected	25	25	29	29
Number of homozygous mutants	2	4	2	1
Number of heterozygous plants	9	13	6	5
Mutation rate (%)	44	68	28	21

.

. **Supplementary Table 2:** Primers used in this study

Primer name	Primers sequence (5'-3')
OsGA20ox2-H1-gRNA1	5'-ACATGCCCGTGGTCGACGTGG-3'
OsGA20ox2-H2-gRNA2	5'-TCCTCCTCCAGGACGACGTCGG-3'
SE5-gRNA3	5'-GCGGCGTGTCCGTGCACGCCGG-3'
OsGA20ox2-1-site-idFP	5'-CAACTCACTCCCGCTCAACACAGC-3'
OsGA20ox2-1-site-idRP	5'-TTTGAAATGCAATGTCGTCCACC-3'
OsGA20ox2-2-site-idFP	5'-GCGCCAATGGGGTAATTAAAACG-3'
OsGA20ox2-2-site-idRP	5'-GGCATTCCATTGTTTGTGATTGG-3'
SE5-site-idFP	5'-ACTCCTCACTCCGCAGAAGC-3'
SE5-site-idRP	5'-GATCACTCACACCAGGGGAC-3'
Pi54- checkFP	5'-CATGAGTTCCATTTACTATTCCTC-3'
Pi54- checkRP	5'-ACATTGGTAGTAGTGCAATGTCA-3'
Rc-bHLH-checkFP	5'-CCATTTCCCTTCTGTCACAG-3'
Rc-bHLH-checkRP	5'-AAAGGTACCAAAGATCGCAG-3'
Ghd8check-FP	5'-CAACTCCCATAACCTCCCC-3'
Ghd8check-RP	5'-CAACCGAACTCCTACAGAAA-3'
HD1-check- FP	5'-TACTACCACAAGCAAGGCTA-3'
HD1-check- RP	5'-CTCCCTTCCTTCTCTGCAA-3'
$S5^{N}$- check- FP	5'-CGTCTTGCTTCTTCATTCCC-3'
$S5^{N}$- check- RP	5'-GTAGGTAAACACAGGCAGAG-3'
OsGSK1 -check- FP	5'-GGTAAAACCACTTCCCCATC-3'
OsGSK1 -check- RP	5'-AACTGTATCTAGTCAGCCCC-3'
